# Supplementary material for: Changes in the associations of race and rurality with SARS-CoV-2 infection, mortality, and case fatality in the United States from February 2020 to March 2021: A population-based cohort study
Source: PLoS Med. 2021 Oct 21;18(10):e1003807. doi: 10.1371/journal.pmed.1003807 (PMC8530298; doi:10.1371/journal.pmed.1003807)
Supplement: S1 Table — AOR, adjusted odds ratio; SARS-CoV-2, Severe Acute Respiratory Syndrome Coronavirus 2; VA, Veterans Affairs. (DOCX) [file pmed.1003807.s002.docx]

**S1 Table. Adjusted* odds ratio for interaction term of risk factor and monthly time period of SARS-CoV-2 infection treated as an ordinal variable (risk factor * time period) among 9.1 million VA enrollees from February 2020 to March 2021, performed as a test of trends over time**

|  | **Adjusted* Odd Ratio for SARS-CoV-2 infection** | **P-value**** |
| --- | --- | --- |
| **Sex** |  | 0.04 |
| Female | 1 | - |
| Male | 1.003 | 0.04 |
| **Age (years)** |  | < 0.001 |
| 18-64 | 1 | - |
| 65-79 | 1.011 | < 0.001 |
| ≥ 80 | 0.998 | 0.33 |
| **Race** |  | < 0.001 |
| White | 1 | - |
| Black | 0.934 | < 0.001 |
| Asian | 0.996 | 0.45 |
| AI/AN | 0.977 | < 0.001 |
| PI/NH | 0.986 | < 0.01 |
| Missing/  Unknown/  Refused | 0.983 | < 0.001 |
| **Ethnicity** |  | < 0.001 |
| Non-Hispanic | 1 | - |
| Hispanic | 0.972 | < 0.001 |
| Missing/  Unknown/  Refused | 1.009 | < 0.01 |
| **US Federal Region†** |  | < 0.001 |
| 1 | 0.985 | < 0.001 |
| 2 | 0.937 | < 0.001 |
| 3 | 1.023 | < 0.001 |
| 4 | 1 | - |
| 5 | 1.005 | < 0.01 |
| 6 | 0.978 | < 0.001 |
| 7 | 1.015 | < 0.001 |
| 8 | 1.01 | < 0.001 |
| 9 | 1.026 | < 0.001 |
| 10 | 1.008 | < 0.01 |
| **Urban vs. Rural** |  | < 0.001 |
| Rural | 1 | - |
| Urban | 0.957 | < 0.001 |
| **BMI (kg/m^2^)** |  | < 0.001 |
| <18.5  (Underweight) | 0.979 | < 0.001 |
| 18.5 to <25  (Normal-weight) | 1 | - |
| 25 to <30  (Over-weight) | 1.011 | < 0.001 |
| 30 to <35  (Obese I) | 1.015 | < 0.001 |
| 35 to <40  (Obese II) | 1.015 | < 0.001 |
| ≥40  (Obese III) | 1.016 | < 0.001 |
| **Charlson Comorbidity Index  (CCI)** |  | < 0.001 |
| 0-1 | 1 | - |
| 2-3 | 1.005 | < 0.001 |
| 4-5 | 0.998 | 0.12 |
| ≥6 | 0.983 | < 0.001 |

* Adjusted for sex, age, race, ethnicity, geographical region, urban/rural location, BMI and CCI.

†Categorized according to the 10 Federal Regions drawn up by the Federal Emergency Management Agency: 1 (CT, MA, ME, NH, RI, VT), 2 (NJ, NY, PR), 3 (DC, DE, MD, PA, VA, WV), 4 (AL, FL, GA, KY, MS, NC, SC, TN), 5 (IL, IN, MI, MN, OH, WI), 6 (AR, LA, NM, OK, TX), 7 (IA, KS, MO, NE), 8 (CO, MT, ND, SD, UT, WY), 9 (AZ, CA, GU, HI, NV), 10 (AK, ID, OR, WA).

** Overall Wald test for all interaction coefficients equal to 0 and Wald test for predictor level and month interaction coefficient equal to 0.
