# Supplementary material for: Changes in the associations of race and rurality with SARS-CoV-2 infection, mortality, and case fatality in the United States from February 2020 to March 2021: A population-based cohort study
Source: PLoS Med. 2021 Oct 21;18(10):e1003807. doi: 10.1371/journal.pmed.1003807 (PMC8530298; doi:10.1371/journal.pmed.1003807)
Supplement: S2 Table — AOR, adjusted odds ratio; SARS-CoV-2, Severe Acute Respiratory Syndrome Coronavirus 2; VA, Veterans Affairs. (DOCX) [file pmed.1003807.s003.docx]

**S2 Table. Adjusted* odds ratio for interaction term of risk factor and monthly time period of SARS-CoV-2-related mortality treated as an ordinal variable (risk factor * time period) among 9.1 million VA enrollees from February 2020 to February 2021, performed as a test of trends over time**

|  | **Adjusted* Odd Ratio for SARS-CoV-2-related Mortality** | **P-value**** |
| --- | --- | --- |
| **Sex** |  | 0.40 |
| Female | 1 | - |
| Male | 1.039 | 0.40 |
| **Age (years)** |  | < 0.01 |
| 18-64 | 1 | - |
| 65-79 | 1.074 | < 0.01 |
| ≥ 80 | 1.052 | 0.04 |
| **Race** |  | < 0.001 |
| White | 1 | - |
| Black | 0.762 | < 0.001 |
| Asian | 0.919 | 0.28 |
| AI/AN | 0.985 | 0.80 |
| PI/NH | 1.044 | 0.57 |
| Missing/  Unknown/  Refused | 0.993 | 0.78 |
| **Ethnicity** |  | 0.02 |
| Non-Hispanic | 1 | - |
| Hispanic | 0.996 | 0.88 |
| Missing/  Unknown/  Refused | 1.11 | < 0.01 |
| **US Federal Region†** |  | < 0.001 |
| 1 | 0.693 | < 0.001 |
| 2 | 0.611 | < 0.001 |
| 3 | 0.956 | 0.10 |
| 4 | 1 | - |
| 5 | 0.946 | < 0.01 |
| 6 | 0.962 | 0.06 |
| 7 | 1.096 | < 0.001 |
| 8 | 1.015 | 0.65 |
| 9 | 1.171 | < 0.001 |
| 10 | 1.002 | 0.96 |
| **Urban vs. Rural** |  | < 0.001 |
| Rural | 1 | - |
| Urban | 0.79 | < 0.001 |
| **BMI (kg/m^2^)** |  | 0.05 |
| <18.5  (Underweight) | 0.986 | 0.72 |
| 18.5 to <25  (Normal-weight) | 1 | - |
| 25 to <30  (Over-weight) | 1.034 | 0.06 |
| 30 to <35  (Obese I) | 1.056 | < 0.01 |
| 35 to <40  (Obese II) | 1.045 | 0.07 |
| ≥40  (Obese III) | 1.006 | 0.82 |
| **Charlson Comorbidity Index  (CCI)** |  | 0.04 |
| 0-1 | 1 | - |
| 2-3 | 1.021 | 0.36 |
| 4-5 | 1.039 | 0.08 |
| ≥6 | 0.992 | 0.68 |

* Adjusted for sex, age, race, ethnicity, geographical region, urban/rural location, BMI and CCI.

†Categorized according to the 10 Federal Regions drawn up by the Federal Emergency Management Agency: 1 (CT, MA, ME, NH, RI, VT), 2 (NJ, NY, PR), 3 (DC, DE, MD, PA, VA, WV), 4 (AL, FL, GA, KY, MS, NC, SC, TN), 5 (IL, IN, MI, MN, OH, WI), 6 (AR, LA, NM, OK, TX), 7 (IA, KS, MO, NE), 8 (CO, MT, ND, SD, UT, WY), 9 (AZ, CA, GU, HI, NV), 10 (AK, ID, OR, WA).

** Overall Wald test for all interaction coefficients equal to 0 and Wald test for predictor level and month interaction coefficient equal to 0.
