# Supplementary material for: Changes in the associations of race and rurality with SARS-CoV-2 infection, mortality, and case fatality in the United States from February 2020 to March 2021: A population-based cohort study
Source: PLoS Med. 2021 Oct 21;18(10):e1003807. doi: 10.1371/journal.pmed.1003807 (PMC8530298; doi:10.1371/journal.pmed.1003807)
Supplement: S3 Table — AOR, adjusted odds ratio;SARS-CoV-2, Severe Acute Respiratory Syndrome Coronavirus 2; VA, Veterans Affairs. (DOCX) [file pmed.1003807.s004.docx]

**S3 Table. Trends over time in the associations (adjusted* odds ratios) of race with risk of SARS-CoV-2 infection presented separately for persons aged <65 versus ≥65 year among 9.1 million VA enrollees from February 2020 to March 2021.**

|  | **Adjusted* Odd Ratio for SARS-CoV-2 infection (95% CI)** | | | | | | | | | | | | | |
| --- | --- | --- | --- | --- | --- | --- | --- | --- | --- | --- | --- | --- | --- | --- |
|  | **Entire Period: February 2020-March 2021**  **N=**9,127,673 | **February- March 2020**  **N=**9,127,673 | **April**  **2020**  **N=**9,090,196 | **May**  **2020**  **N=**9,053,082 | **June**  **2020**  **N=**9,022,051 | **July**  **2020**  **N=**8,990,833 | **August**  **2020**  **N=**8,948,674 | **September 2020**  **N=**8,913,864 | **October**  **2020**  **N=**8,881,573 | **November 2020**  **N=**8,844,680 | **December**  **2020**  **N=**8,804,424 | **January**  **2021**  **N=**8,744,388 | **February**  **2021**  **N=**8,671,916 | **March**  **2021**  **N=**8,630,283 |
| **Age < 65 years** |  |  |  |  |  |  |  |  |  |  |  |  |  |  |
| **Race** |  |  |  |  |  |  |  |  |  |  |  |  |  |  |
| White | 1 | 1 | 1 | 1 | 1 | 1 | 1 | 1 | 1 | 1 | 1 | 1 | 1 | 1 |
| Black | 1.36 (1.34-1.38) | 4.05 (3.58-4.59) | 3.06 (2.83-3.31) | 2.77 (2.52-3.05) | 1.77 (1.65-1.90) | 1.86 (1.78-1.95) | 1.74 (1.63-1.86) | 1.36 (1.26-1.47) | 1.13 (1.06-1.20) | 1.02 (0.98-1.06) | 1.13 (1.09-1.16) | 1.25 (1.21-1.30) | 1.29 (1.23-1.36) | 1.14 (1.07-1.22) |
| Asian | 0.93 (0.89-0.98) | 1.75 (1.12-2.72) | 1.32 (0.97-1.79) | 1.15 (0.79-1.67) | 0.82 (0.64-1.06) | 0.94 (0.79-1.12) | 0.90 (0.69-1.17) | 1.02 (0.77-1.34) | 0.81 (0.65-1.02) | 0.83 (0.73-0.95) | 0.92 (0.84-1.02) | 0.98 (0.88-1.10) | 1.02 (0.86-1.20) | 0.81 (0.64-1.03) |
| AI/AN | 0.88 (0.83-0.94) | 0.59 (0.24-1.43) | 1.20 (0.82-1.75) | 1.74 (1.21-2.50) | 1.23 (0.96-1.58) | 0.92 (0.75-1.12) | 0.84 (0.62-1.12) | 0.99 (0.73-1.33) | 0.94 (0.76-1.17) | 0.88 (0.77-1.02) | 0.83 (0.73-0.94) | 0.83 (0.72-0.96) | 0.86 (0.69-1.06) | 0.68 (0.50-0.93) |
| PI/NH | 0.97 (0.92-1.03) | 1.15 (0.61-2.15) | 1.56 (1.12-2.17) | 1.40 (0.94-2.11) | 1.07 (0.83-1.39) | 0.96 (0.79-1.17) | 1.25 (0.97-1.61) | 0.84 (0.59-1.18) | 0.93 (0.73-1.19) | 0.86 (0.74-1.00) | 0.95 (0.85-1.07) | 0.96 (0.85-1.10) | 1.08 (0.89-1.31) | 0.76 (0.57-1.03) |
| Missing/  Unknown/  Refused | 0.87 (0.85-0.89) | 1.17 (0.91-1.50) | 1.13 (0.97-1.32) | 1.18 (0.99-1.42) | 0.88 (0.78-0.99) | 0.88 (0.81-0.96) | 0.93 (0.82-1.04) | 0.91 (0.80-1.05) | 0.84 (0.75-0.93) | 0.82 (0.77-0.87) | 0.86 (0.82-0.91) | 0.88 (0.83-0.93) | 0.86 (0.79-0.94) | 0.82 (0.73-0.93) |
| **Age ≥ 65 years** |  |  |  |  |  |  |  |  |  |  |  |  |  |  |
| **Race** |  |  |  |  |  |  |  |  |  |  |  |  |  |  |
| White | 1 | 1 | 1 | 1 | 1 | 1 | 1 | 1 | 1 | 1 | 1 | 1 | 1 | 1 |
| Black | 1.46 (1.43-1.49) | 6.57 (5.76-7.50) | 3.15 (2.93-3.39) | 2.34 (2.14-2.57) | 2.24 (2.06-2.45) | 1.98 (1.86-2.11) | 1.77 (1.64-1.92) | 1.29 (1.18-1.42) | 0.95 (0.88-1.04) | 1.06 (1.01-1.12) | 1.19 (1.14-1.24) | 1.27 (1.22-1.33) | 1.31 (1.23-1.39) | 1.25 (1.14-1.37) |
| Asian | 0.59 (0.54-0.65) | 1.59 (0.70-3.60) | 0.62 (0.34-1.12) | 0.62 (0.31-1.25) | 0.45 (0.26-0.78) | 0.75 (0.53-1.04) | 0.92 (0.60-1.41) | 0.94 (0.58-1.53) | 0.66 (0.41-1.05) | 0.47 (0.34-0.64) | 0.57 (0.47-0.69) | 0.52 (0.42-0.64) | 0.61 (0.45-0.83) | 0.51 (0.29-0.87) |
| AI/AN | 1.03 (0.96-1.10) | 2.10 (1.08-4.07) | 1.58 (1.08-2.32) | 1.28 (0.80-2.04) | 1.13 (0.77-1.66) | 1.00 (0.77-1.31) | 0.98 (0.70-1.38) | 1.02 (0.72-1.46) | 1.09 (0.85-1.40) | 1.03 (0.87-1.22) | 0.96 (0.83-1.12) | 0.98 (0.83-1.15) | 1.15 (0.91-1.44) | 0.61 (0.38-0.97) |
| PI/NH | 0.97 (0.90-1.04) | 0.61 (0.20-1.91) | 1.00 (0.65-1.54) | 1.26 (0.81-1.96) | 0.68 (0.43-1.08) | 0.92 (0.70-1.22) | 1.16 (0.84-1.60) | 1.52 (1.11-2.07) | 1.02 (0.77-1.36) | 1.14 (0.96-1.36) | 0.75 (0.63-0.88) | 0.99 (0.84-1.15) | 1.02 (0.80-1.29) | 0.82 (0.55-1.23) |
| Missing/  Unknown/  Refused | 0.73 (0.71-0.76) | 1.34 (0.98-1.82) | 0.93 (0.78-1.10) | 1.03 (0.85-1.25) | 0.72 (0.60-0.87) | 0.87 (0.77-0.97) | 0.73 (0.62-0.85) | 0.79 (0.67-0.93) | 0.73 (0.64-0.83) | 0.77 (0.71-0.83) | 0.71 (0.67-0.76) | 0.68 (0.63-0.74) | 0.67 (0.60-0.75) | 0.65 (0.55-0.77) |

* Adjusted for sex, age, race, ethnicity, geographical region, urban/rural location, BMI and CCI.

†Categorized according to the 10 Federal Regions drawn up by the Federal Emergency Management Agency: 1 (CT, MA, ME, NH, RI, VT), 2 (NJ, NY, PR), 3 (DC, DE, MD, PA, VA, WV), 4 (AL, FL, GA, KY, MS, NC, SC, TN), 5 (IL, IN, MI, MN, OH, WI), 6 (AR, LA, NM, OK, TX), 7 (IA, KS, MO, NE), 8 (CO, MT, ND, SD, UT, WY), 9 (AZ, CA, GU, HI, NV), 10 (AK, ID, OR, WA).
