# Supplementary material for: Changes in the associations of race and rurality with SARS-CoV-2 infection, mortality, and case fatality in the United States from February 2020 to March 2021: A population-based cohort study
Source: PLoS Med. 2021 Oct 21;18(10):e1003807. doi: 10.1371/journal.pmed.1003807 (PMC8530298; doi:10.1371/journal.pmed.1003807)
Supplement: S1 Analytic Plan — SARS-CoV-2, Severe Acute Respiratory Syndrome Coronavirus 2. (DOCX) [file pmed.1003807.s005.docx]

**S1 Analytic Plan**

**Changes in risk factors for SARS-CoV-2 infection and mortality over time in a national healthcare system**

**Introduction**

Sociodemographic factors and comorbidity burden have emerged as major risk factors for SARS-CoV-2 infection and mortality(1-13). Black, American Indian/Alaska Native (AI/AN) and Hispanic persons have been reported to have higher risk of SARS-CoV-2 infection and mortality than White and non-Hispanic persons(7-11, 14). Obese persons(12, 15) and those with higher comorbidity burden(9) have also been reported to have higher risk of SARS-CoV-2 infection and mortality, while older age is one of the strongest risk factors for SARS-CoV-2-related mortality(2, 9). Residing in a geographical region with high incidence of SARS-CoV-2 infection at a given time is a strong risk factor for SARS-CoV-2 infection and mortality(2, 5, 16).

Risk factors for SARS-CoV-2-related infection and mortality may be changing over time since the pandemic began. Although it is clear that geographical regions with high infection, hospitalization and mortality rates changed over time as the pandemic surged in different parts of the country, key risk factors such as age, comorbidity burden, race and ethnicity may also change due to societal changes in prophylactic measures, disease awareness and behavior, and access to care. Known and unknown viral factors (e.g. emergence of new variants) and treatment factors (e.g. use of different pharmacotherapies) are also changing over time and may result in changes in the associations of certain risk factors with infection or mortality. Recognizing the changing patterns of risk factors is important to support population-based approaches to prevent infection and reduce mortality by helping target those at highest risk during an evolving pandemic.

We identified a cohort of xxx million persons who were enrolled in the national United States Department of Veterans Affairs (VA) healthcare system at the beginning of the pandemic in February 2020 and followed this cohort over the ensuing 8-month period to determine whether established risk factors for SARS-CoV-2 infection and mortality changed over time.

**Methods**

**Study population and data source**

The VA supports the largest integrated national healthcare system in the United States, providing care at 168 medical centers and 1,053 outpatient clinics throughout the country. We identified a cohort of all persons aged ≥18 years who were alive and enrolled in the VA healthcare system on February 1, 2020 (n=xxxx). We followed this cohort for the development of SARS-CoV-2 infection and SARS-CoV-2-related mortality until October 31, 2020.

The VA employs a comprehensive, nationwide electronic health records system (EHR). Patient EHR data from all VA facilities are transferred to the VA’s centralized relational database, the Corporate Data Warehouse (CDW), on a nightly basis to support and streamline research and clinical operations activities(17). The CDW includes the “COVID-19 Shared Data Resource”, a set of analytic variables and datasets related to COVID-19 developed and maintained by the VA Informatics and Computing Infrastructure (VINCI) specifically to facilitate COVID-19 research and operations, which we used in combination with all CDW data for the current study.

The study was approved by the VA Puget Sound Institutional Review Board.

**Definition of SARS-CoV-2 infection and SARS-CoV-2-related death.**

Cohort members who tested positive for SARS-CoV-2 within the VA system based on polymerase chain reaction (PCR) tests were defined as having infection. The earliest date of a documented positive test was taken as each patient’s date of infection. Patients who died of any cause within 30 days of infection were defined as having a SARS-CoV-2-related death consistent with prior studies (2, 3) Deaths occurring both within and outside the VA are comprehensively captured in CDW from a variety of VA and non-VA sources including VA inpatient files, VA Beneficiary Identification and Records Locator System (BIRLS), Social Security Administration (SSA) death files, and the Department of Defense (18). Deaths occurring from February 1 to October 31, 2020 were ascertained from files that were updated on December 15, 2020 to allow time for deaths to be electronically recorded in CDW.

SARS-CoV-2 infection and mortality rates were calculated monthly as a proportion of all VA enrollees in our cohort who were still alive and at risk at the beginning of each month. Mortality was also calculated bimonthly because there were too few deaths in some subgroups to generate reasonably precise monthly analyses of time trends. For the monthly analysis of SARS-CoV-2 infection rates we excluded persons who died of any cause or were infected with SARS-CoV-2 before the beginning of each monthly period. For the analysis of SARS-CoV-2 mortality rates we excluded persons who died of any cause before the beginning of each monthly period or were infected with SARS-CoV-2 more than 30 days before the beginning of the month (since SARS-CoV-2-related mortality was defined as death within 30 days of infection). The aim of removing from our cohort persons who were no longer at risk at the beginning of each observation period was to avoid a spurious attenuation of risk factors over time due to “depletion” of at-risk persons.

**Sociodemographic factors and comorbidity burden.**

We ascertained the following 8 characteristics for all cohort members on the first day of each monthly observation period: age, sex, self-reported race (White, Black, Asian, American Indian/Alaska Native [AI/AN], Pacific Islander/Native Hawaiian [PI/NH], Other) and ethnicity (Hispanic, non-Hispanic), urban vs rural residence (based on zip codes, using data from the VA Office of Rural Health(19), which uses the Secondary Rural-Urban Commuting Area [RUCA] for defining rurality), geographic location (divided into 10 standard U.S. Federal Regions(20)), body mass index (BMI, categorized using the World Health Organization groups(21) as shown in Table A), and Charlson Comorbidity Index (CCI) (22) calculated using the Deyo modification(23), which takes into account 19 comorbid conditions reported in the two years prior to the observation period. We focused on these sociodemographic factors, obesity and the CCI because they are some of the most important risk factors for SARS-CoV-2 infection or mortality reported in the literature(1-13) and because trends in the associations over time could be plausibly hypothesized.

**Statistical Analysis.**

We calculated the number of new SARS-CoV-2 infections and deaths each month and the monthly incidence as a proportion of all persons in our cohort who were still alive and at risk on the first day of the month. For each monthly or bimonthly observation period, we used multivariable logistic regression simultaneously adjusting for the 8 characteristics listed above to determine the associations between each risk factor and SARS-CoV-2 infection or death, reported as the adjusted odds ratio.

We used a Wald test with cluster-robust standard errors to formally evaluate whether the associations between risk factors and each outcome (infection or death) changed over time by creating a model that combined all time periods and including an interaction term separately for each risk factor (risk factor*time period) where time period was an ordinal variable.

**Results**

**References**

1. Williamson EJ, Walker AJ, Bhaskaran K, Bacon S, Bates C, Morton CE, et al. Factors associated with COVID-19-related death using OpenSAFELY. Nature. 2020;584(7821):430-6.

2. Ioannou GN, Locke E, Green P, Berry K, O'Hare AM, Shah JA, et al. Risk Factors for Hospitalization, Mechanical Ventilation, or Death Among 10131 US Veterans With SARS-CoV-2 Infection. JAMA Netw Open. 2020;3(9):e2022310.

3. King JT, Jr., Yoon JS, Rentsch CT, Tate JP, Park LS, Kidwai-Khan F, et al. Development and validation of a 30-day mortality index based on pre-existing medical administrative data from 13,323 COVID-19 patients: The Veterans Health Administration COVID-19 (VACO) Index. PLoS One. 2020;15(11):e0241825.

4. Rentsch CT, Kidwai-Khan F, Tate JP, Park LS, King JT, Jr., Skanderson M, et al. Patterns of COVID-19 testing and mortality by race and ethnicity among United States veterans: A nationwide cohort study. PLoS Med. 2020;17(9):e1003379.

5. Fan VS, Dominitz JA, Eastment MC, Locke E, Green P, Berry K, et al. Risk Factors for testing positive for SARS-CoV-2 in a national US healthcare system. Clin Infect Dis. 2020.

6. Dai CL, Kornilov SA, Roper RT, Cohen-Cline H, Jade K, Smith B, et al. Characteristics and Factors Associated with COVID-19 Infection, Hospitalization, and Mortality Across Race and Ethnicity. Clin Infect Dis. 2021.

7. Bassett MT, Chen JT, Krieger N**.** Variation in racial/ethnic disparities in COVID-19 mortality by age in the United States: A cross-sectional study. PLoS Med. 2020;17(10):e1003402.

8. Gold JAW, Rossen LM, Ahmad FB, Sutton P, Li Z, Salvatore PP, et al. Race, Ethnicity, and Age Trends in Persons Who Died from COVID-19 - United States, May-August 2020. MMWR Morb Mortal Wkly Rep. 2020;69(42):1517-21.

9. Escobar GJ, Adams AS, Liu VX, Soltesz L, Chen YI, Parodi SM, et al. Racial Disparities in COVID-19 Testing and Outcomes : Retrospective Cohort Study in an Integrated Health System. Ann Intern Med. 2021.

10. Mackey K, Ayers CK, Kondo KK, Saha S, Advani SM, Young S, et al. Racial and Ethnic Disparities in COVID-19-Related Infections, Hospitalizations, and Deaths : A Systematic Review. Ann Intern Med. 2021;174(3):362-73.

11. Price-Haywood EG, Burton J, Fort D, Seoane L**.** Hospitalization and Mortality among Black Patients and White Patients with Covid-19. N Engl J Med. 2020;382(26):2534-43.

12. Eastment MC, Berry K, Locke E, Green P, O'Hare A, Crothers K, et al. Body mass index (BMI) and outcomes of SARS-CoV-2 among US veterans. Obesity (Silver Spring). 2020.

13. Ioannou GN, Green P, Fan VS, Dominitz JA, O'Hare AM, Backus LI, et al. Development of COVIDVax Model to Estimate the Risk of SARS-CoV-2-Related Death Among 7.6 Million US Veterans for Use in Vaccination Prioritization. JAMA Netw Open. 2021;4(4):e214347.

14. Ferguson JM, Abdel Magid HS, Purnell AL, Kiang MV, Osborne TF**.** Differences in COVID-19 Testing and Test Positivity Among Veterans, United States, 2020. Public Health Rep. 2021:333549211009498.

15. Tartof SY, Qian L, Hong V, Wei R, Nadjafi RF, Fischer H, et al. Obesity and Mortality Among Patients Diagnosed With COVID-19: Results From an Integrated Health Care Organization. Ann Intern Med. 2020;173(10):773-81.

16. Asch DA, Sheils NE, Islam MN, Chen Y, Werner RM, Buresh J, et al. Variation in US Hospital Mortality Rates for Patients Admitted With COVID-19 During the First 6 Months of the Pandemic. JAMA Intern Med. 2020.

17. Veterans Affairs Corporate Data Warehouse. Available at: <http://www.hsrd.research.va.gov/for_researchers/vinci/cdw.cfm> Last accessed on 03/22/21.

18. Sohn MW, Arnold N, Maynard C, Hynes DM**.** Accuracy and completeness of mortality data in the Department of Veterans Affairs. Popul Health Metr. 2006;4:2.

19. Veterans Health Administration, Office of Rural Health. Available at: <https://vaww.vashare.vha.va.gov/sites/ruralhealth/GSOD/default.aspx> Last accessed 03/28/2021.

20. Federal Emergency Management (FEMA) US Regions. Available at: <https://www.fema.gov/about/organization/regions> Last accessed 02/12/2021.

21. World Health Organization. Body Mass Index. Available at: <https://www.euro.who.int/en/health-topics/disease-prevention/nutrition/a-healthy-lifestyle/body-mass-index-bmi> Last accessed on 03/21/2021.

22. Charlson ME, Pompei P, Ales KL, MacKenzie CR**.** A new method of classifying prognostic comorbidity in longitudinal studies: development and validation. J Chronic Dis. 1987;40(5):373-83.

23. Deyo RA, Cherkin DC, Ciol MA**.** Adapting a clinical comorbidity index for use with ICD-9-CM administrative databases. J Clin Epidemiol. 1992;45(6):613-9.

**Fig A.** Trends over time in the associations of the following factors with risk of SARS-CoV-2 infection and mortality:

A & B. Black vs. White race

C & D. Urban vs. rural location

E & F. CCI categories

G & H. Age categories

**Table A. Cohort characteristics and incidence of SARS-CoV-2 infection presented by month in a cohort of xxxx million VA enrollees followed from February, 2020 to March, 2021**

|  | **Cohort Characteristics**  **N (%)** | **Number of SARS-CoV-2 positive tests (and incidence per 10,000 persons per month)** | | | | | | | | | | | | |
| --- | --- | --- | --- | --- | --- | --- | --- | --- | --- | --- | --- | --- | --- | --- |
| **Time Period and number of VA enrollees at risk*** | **Entire Period: February, 2020-March, 2021**  **N=** | **February- March, 2020**  **N=** | **April, 2020**  **N=** | **May, 2020**  **N=** | **June, 2020**  **N=** | **July, 2020**  **N=** | **August, 2020**  **N=** | **September, 2020**  **N=** | **October, 2020**  **N=** | **November, 2020**  **N=** | **December, 2020**  **N=** | **January, 2021**  **N=** | **February, 2021**  **N=** | **March, 2021**  **N=** |
| **All Persons** |  |  |  |  |  |  |  |  |  |  |  |  |  |  |
| **Sex** |  |  |  |  |  |  |  |  |  |  |  |  |  |  |
| Female |  |  |  |  |  |  |  |  |  |  |  |  |  |  |
| Male |  |  |  |  |  |  |  |  |  |  |  |  |  |  |
| **Age (years)** |  |  |  |  |  |  |  |  |  |  |  |  |  |  |
| 18-64 |  |  |  |  |  |  |  |  |  |  |  |  |  |  |
| 65-79 |  |  |  |  |  |  |  |  |  |  |  |  |  |  |
| ≥ 80 |  |  |  |  |  |  |  |  |  |  |  |  |  |  |
| **Race** |  |  |  |  |  |  |  |  |  |  |  |  |  |  |
| White |  |  |  |  |  |  |  |  |  |  |  |  |  |  |
| Black |  |  |  |  |  |  |  |  |  |  |  |  |  |  |
| Asian |  |  |  |  |  |  |  |  |  |  |  |  |  |  |
| American Indian/ Alaska Native |  |  |  |  |  |  |  |  |  |  |  |  |  |  |
| Pacific Islander/ Native Hawaiian |  |  |  |  |  |  |  |  |  |  |  |  |  |  |
| Missing/Unknown/Refused |  |  |  |  |  |  |  |  |  |  |  |  |  |  |
| **Ethnicity** |  |  |  |  |  |  |  |  |  |  |  |  |  |  |
| Non-Hispanic |  |  |  |  |  |  |  |  |  |  |  |  |  |  |
| Hispanic |  |  |  |  |  |  |  |  |  |  |  |  |  |  |
| Missing/Unknown/Refused |  |  |  |  |  |  |  |  |  |  |  |  |  |  |
| **US Federal Region†** |  |  |  |  |  |  |  |  |  |  |  |  |  |  |
| 1 |  |  |  |  |  |  |  |  |  |  |  |  |  |  |
| 2 |  |  |  |  |  |  |  |  |  |  |  |  |  |  |
| 3 |  |  |  |  |  |  |  |  |  |  |  |  |  |  |
| 4 |  |  |  |  |  |  |  |  |  |  |  |  |  |  |
| 5 |  |  |  |  |  |  |  |  |  |  |  |  |  |  |
| 6 |  |  |  |  |  |  |  |  |  |  |  |  |  |  |
| 7 |  |  |  |  |  |  |  |  |  |  |  |  |  |  |
| 8 |  |  |  |  |  |  |  |  |  |  |  |  |  |  |
| 9 |  |  |  |  |  |  |  |  |  |  |  |  |  |  |
| 10 |  |  |  |  |  |  |  |  |  |  |  |  |  |  |
| **Urban vs. Rural** |  |  |  |  |  |  |  |  |  |  |  |  |  |  |
| Rural/Highly rural |  |  |  |  |  |  |  |  |  |  |  |  |  |  |
| Urban |  |  |  |  |  |  |  |  |  |  |  |  |  |  |
| **BMI (kg/m^2^)** |  |  |  |  |  |  |  |  |  |  |  |  |  |  |
| <18.5 (Underweight) |  |  |  |  |  |  |  |  |  |  |  |  |  |  |
| 18.5 to <25 (Normal-weight) |  |  |  |  |  |  |  |  |  |  |  |  |  |  |
| 25 to <30 (Over-weight) |  |  |  |  |  |  |  |  |  |  |  |  |  |  |
| 30 to <35 (Obese I) |  |  |  |  |  |  |  |  |  |  |  |  |  |  |
| 35 to <40 (Obese II) |  |  |  |  |  |  |  |  |  |  |  |  |  |  |
| ≥40 (Obese III) |  |  |  |  |  |  |  |  |  |  |  |  |  |  |
| **Charlson Comorbidity Index (CCI)** |  |  |  |  |  |  |  |  |  |  |  |  |  |  |
| 0-1 |  |  |  |  |  |  |  |  |  |  |  |  |  |  |
| 2-3 |  |  |  |  |  |  |  |  |  |  |  |  |  |  |
| 4-5 |  |  |  |  |  |  |  |  |  |  |  |  |  |  |
| ≥6 |  |  |  |  |  |  |  |  |  |  |  |  |  |  |

* VA enrollees at risk are those who are still alive and not yet infected at the beginning of each time period

†Categorized according to the 10 US Federal Regions drawn up by the Office of Management and Budget: 1 (CT, MA, ME, NH, RI, VT), 2 (NJ, NY, PR, Virgin Island), 3 (DC, DE, MD, PA, VA, WV), 4 (AL, FL, GA, KY, MS, NC, SC, TN), 5 (IL, IN, MI, MN, OH, WI), 6 (AR, LA, NM, OK, TX), 7 (IA, KS, MO, NE), 8 (CO, MT, ND, SD, UT, WY), 9 (AZ, CA, GU, HI, NV), 10 (AK, ID, OR, WA).

Table B. Trends over time in the associations (adjusted* odds ratios) of sociodemographic characteristics and comorbidity burden **with risk of SARS-CoV-2 infection** among all xxxx VA enrollees

|  | **Adjusted* Odd Ratio for SARS-CoV-2 infection (95% CI)** | | | | | | | | | | | | | |
| --- | --- | --- | --- | --- | --- | --- | --- | --- | --- | --- | --- | --- | --- | --- |
|  | **Entire Period: February, 2020-March, 2021** | **February- March, 2020** | **April, 2020** | **May, 2020** | **June, 2020** | **July, 2020** | **August, 2020** | **September, 2020** | **October, 2020** | **November, 2020** | **December, 2020** | **January, 2021** | **February, 2021** | **March, 2021** |
| **Sex** |  |  |  |  |  |  |  |  |  |  |  |  |  |  |
| Female |  |  |  |  |  |  |  |  |  |  |  |  |  |  |
| Male |  |  |  |  |  |  |  |  |  |  |  |  |  |  |
| **Age (years)** |  |  |  |  |  |  |  |  |  |  |  |  |  |  |
| 18-64 |  |  |  |  |  |  |  |  |  |  |  |  |  |  |
| 65-79 |  |  |  |  |  |  |  |  |  |  |  |  |  |  |
| ≥ 80 |  |  |  |  |  |  |  |  |  |  |  |  |  |  |
| **Race** |  |  |  |  |  |  |  |  |  |  |  |  |  |  |
| White |  |  |  |  |  |  |  |  |  |  |  |  |  |  |
| Black |  |  |  |  |  |  |  |  |  |  |  |  |  |  |
| Asian |  |  |  |  |  |  |  |  |  |  |  |  |  |  |
| American Indian/ Alaska Native |  |  |  |  |  |  |  |  |  |  |  |  |  |  |
| Pacific Islander/ Native Hawaiian |  |  |  |  |  |  |  |  |  |  |  |  |  |  |
| Missing/Unknown/Refused |  |  |  |  |  |  |  |  |  |  |  |  |  |  |
| **Ethnicity** |  |  |  |  |  |  |  |  |  |  |  |  |  |  |
| Non-Hispanic |  |  |  |  |  |  |  |  |  |  |  |  |  |  |
| Hispanic |  |  |  |  |  |  |  |  |  |  |  |  |  |  |
| Missing/Unknown/Refused |  |  |  |  |  |  |  |  |  |  |  |  |  |  |
| **US Federal Region†** |  |  |  |  |  |  |  |  |  |  |  |  |  |  |
| 1 |  |  |  |  |  |  |  |  |  |  |  |  |  |  |
| 2 |  |  |  |  |  |  |  |  |  |  |  |  |  |  |
| 3 |  |  |  |  |  |  |  |  |  |  |  |  |  |  |
| 4 |  |  |  |  |  |  |  |  |  |  |  |  |  |  |
| 5 |  |  |  |  |  |  |  |  |  |  |  |  |  |  |
| 6 |  |  |  |  |  |  |  |  |  |  |  |  |  |  |
| 7 |  |  |  |  |  |  |  |  |  |  |  |  |  |  |
| 8 |  |  |  |  |  |  |  |  |  |  |  |  |  |  |
| 9 |  |  |  |  |  |  |  |  |  |  |  |  |  |  |
| 10 |  |  |  |  |  |  |  |  |  |  |  |  |  |  |
| **Urban vs. Rural** |  |  |  |  |  |  |  |  |  |  |  |  |  |  |
| Rural/Highly rural |  |  |  |  |  |  |  |  |  |  |  |  |  |  |
| Urban |  |  |  |  |  |  |  |  |  |  |  |  |  |  |
| **BMI (kg/m^2^)** |  |  |  |  |  |  |  |  |  |  |  |  |  |  |
| <18.5 (Underweight) |  |  |  |  |  |  |  |  |  |  |  |  |  |  |
| 18.5 to <25 (Normal-weight) |  |  |  |  |  |  |  |  |  |  |  |  |  |  |
| 25 to <30 (Over-weight) |  |  |  |  |  |  |  |  |  |  |  |  |  |  |
| 30 to <35 (Obese I) |  |  |  |  |  |  |  |  |  |  |  |  |  |  |
| 35 to <40 (Obese II) |  |  |  |  |  |  |  |  |  |  |  |  |  |  |
| ≥40 (Obese III) |  |  |  |  |  |  |  |  |  |  |  |  |  |  |
| **Charlson Comorbidity Index (CCI)** |  |  |  |  |  |  |  |  |  |  |  |  |  |  |
| 0-1 |  |  |  |  |  |  |  |  |  |  |  |  |  |  |
| 2-3 |  |  |  |  |  |  |  |  |  |  |  |  |  |  |
| 4-5 |  |  |  |  |  |  |  |  |  |  |  |  |  |  |
| ≥6 |  |  |  |  |  |  |  |  |  |  |  |  |  |  |

* Adjusted for sex, age, race, ethnicity, geographical region, urban/rural location, BMI and CCI.

†Categorized according to the 10 Federal Regions drawn up by the Federal Emergency Management Agency: 1 (CT, MA, ME, NH, RI, VT), 2 (NJ, NY, PR), 3 (DC, DE, MD, PA, VA, WV), 4 (AL, FL, GA, KY, MS, NC, SC, TN), 5 (IL, IN, MI, MN, OH, WI), 6 (AR, LA, NM, OK, TX), 7 (IA, KS, MO, NE), 8 (CO, MT, ND, SD, UT, WY), 9 (AZ, CA, GU, HI, NV), 10 (AK, ID, OR, WA).

**Table C. SARS-CoV-2-related mortality presented by month in a cohort of xxxx million VA enrollees followed from February, 2020 to March, 2021**

|  | **Number of SARS-CoV-2-related deaths per month (and mortality per 100,000 persons per month)** | | | | | | | | | | | | |
| --- | --- | --- | --- | --- | --- | --- | --- | --- | --- | --- | --- | --- | --- |
| **Time Period and number of VA enrollees at risk*** | **Entire Period: February, 2020-March, 2021**  **N=** | **February- March, 2020**  **N=** | **April, 2020**  **N=** | **May, 2020**  **N=** | **June, 2020**  **N=** | **July, 2020**  **N=** | **August, 2020**  **N=** | **September, 2020**  **N=** | **October, 2020**  **N=** | **November, 2020**  **N=** | **December, 2020**  **N=** | **January, 2021**  **N=** | **February, 2021**  **N=** |
| **All Persons** |  |  |  |  |  |  |  |  |  |  |  |  |  |
| **Sex** |  |  |  |  |  |  |  |  |  |  |  |  |  |
| Female |  |  |  |  |  |  |  |  |  |  |  |  |  |
| Male |  |  |  |  |  |  |  |  |  |  |  |  |  |
| **Age (years)** |  |  |  |  |  |  |  |  |  |  |  |  |  |
| 18-64 |  |  |  |  |  |  |  |  |  |  |  |  |  |
| 65-79 |  |  |  |  |  |  |  |  |  |  |  |  |  |
| ≥ 80 |  |  |  |  |  |  |  |  |  |  |  |  |  |
| **Race** |  |  |  |  |  |  |  |  |  |  |  |  |  |
| White |  |  |  |  |  |  |  |  |  |  |  |  |  |
| Black |  |  |  |  |  |  |  |  |  |  |  |  |  |
| Asian |  |  |  |  |  |  |  |  |  |  |  |  |  |
| American Indian/ Alaska Native |  |  |  |  |  |  |  |  |  |  |  |  |  |
| Pacific Islander/ Native Hawaiian |  |  |  |  |  |  |  |  |  |  |  |  |  |
| Missing/Unknown/Refused |  |  |  |  |  |  |  |  |  |  |  |  |  |
| **Ethnicity** |  |  |  |  |  |  |  |  |  |  |  |  |  |
| Non-Hispanic |  |  |  |  |  |  |  |  |  |  |  |  |  |
| Hispanic |  |  |  |  |  |  |  |  |  |  |  |  |  |
| Missing/Unknown/Refused |  |  |  |  |  |  |  |  |  |  |  |  |  |
| **US Federal Region†** |  |  |  |  |  |  |  |  |  |  |  |  |  |
| 1 |  |  |  |  |  |  |  |  |  |  |  |  |  |
| 2 |  |  |  |  |  |  |  |  |  |  |  |  |  |
| 3 |  |  |  |  |  |  |  |  |  |  |  |  |  |
| 4 |  |  |  |  |  |  |  |  |  |  |  |  |  |
| 5 |  |  |  |  |  |  |  |  |  |  |  |  |  |
| 6 |  |  |  |  |  |  |  |  |  |  |  |  |  |
| 7 |  |  |  |  |  |  |  |  |  |  |  |  |  |
| 8 |  |  |  |  |  |  |  |  |  |  |  |  |  |
| 9 |  |  |  |  |  |  |  |  |  |  |  |  |  |
| 10 |  |  |  |  |  |  |  |  |  |  |  |  |  |
| **Urban vs. Rural** |  |  |  |  |  |  |  |  |  |  |  |  |  |
| Rural/Highly rural |  |  |  |  |  |  |  |  |  |  |  |  |  |
| Urban |  |  |  |  |  |  |  |  |  |  |  |  |  |
| **BMI (kg/m^2^)** |  |  |  |  |  |  |  |  |  |  |  |  |  |
| <18.5 (Underweight) |  |  |  |  |  |  |  |  |  |  |  |  |  |
| 18.5 to <25 (Normal-weight) |  |  |  |  |  |  |  |  |  |  |  |  |  |
| 25 to <30 (Over-weight) |  |  |  |  |  |  |  |  |  |  |  |  |  |
| 30 to <35 (Obese I) |  |  |  |  |  |  |  |  |  |  |  |  |  |
| 35 to <40 (Obese II) |  |  |  |  |  |  |  |  |  |  |  |  |  |
| ≥40 (Obese III) |  |  |  |  |  |  |  |  |  |  |  |  |  |
| **Charlson Comorbidity Index (CCI)** |  |  |  |  |  |  |  |  |  |  |  |  |  |
| 0-1 |  |  |  |  |  |  |  |  |  |  |  |  |  |
| 2-3 |  |  |  |  |  |  |  |  |  |  |  |  |  |
| 4-5 |  |  |  |  |  |  |  |  |  |  |  |  |  |
| ≥6 |  |  |  |  |  |  |  |  |  |  |  |  |  |

* VA enrollees at risk are those who are still alive at the beginning of each time period and had not been infected with SARS-CoV-2 more than 30 days before the beginning of the time period

†Categorized according to the 10 Federal Regions drawn up by the Federal Emergency Management Agency: 1 (CT, MA, ME, NH, RI, VT), 2 (NJ, NY, PR), 3 (DC, DE, MD, PA, VA, WV), 4 (AL, FL, GA, KY, MS, NC, SC, TN), 5 (IL, IN, MI, MN, OH, WI), 6 (AR, LA, NM, OK, TX), 7 (IA, KS, MO, NE), 8 (CO, MT, ND, SD, UT, WY), 9 (AZ, CA, GU, HI, NV), 10 (AK, ID, OR, WA).

Table D. Trends over time in the associations (adjusted odds ratios*) of sociodemographic characteristics and comorbidity burden **with risk of SARS-CoV-2-related mortality** among all xxxx million VA enrollees

|  | **Adjusted* Odd Ratio for SARS-CoV-2-related Mortality (95% CI)** | | | | | | | | | | | | |
| --- | --- | --- | --- | --- | --- | --- | --- | --- | --- | --- | --- | --- | --- |
|  | **Entire Period: February, 2020-March, 2021** | **February- March, 2020** | **April, 2020** | **May, 2020** | **June, 2020** | **July, 2020** | **August, 2020** | **September, 2020** | **October, 2020** | **November, 2020** | **December, 2020** | **January, 2021** | **February, 2021** |
|  |  |  |  |  |  |  |  |  |  |  |  |  |  |
| **Sex** |  |  |  |  |  |  |  |  |  |  |  |  |  |
| Female |  |  |  |  |  |  |  |  |  |  |  |  |  |
| Male |  |  |  |  |  |  |  |  |  |  |  |  |  |
| **Age (years)** |  |  |  |  |  |  |  |  |  |  |  |  |  |
| 18-64 |  |  |  |  |  |  |  |  |  |  |  |  |  |
| 65-79 |  |  |  |  |  |  |  |  |  |  |  |  |  |
| ≥ 80 |  |  |  |  |  |  |  |  |  |  |  |  |  |
| **Race** |  |  |  |  |  |  |  |  |  |  |  |  |  |
| White |  |  |  |  |  |  |  |  |  |  |  |  |  |
| Black |  |  |  |  |  |  |  |  |  |  |  |  |  |
| Asian |  |  |  |  |  |  |  |  |  |  |  |  |  |
| American Indian/ Alaska Native |  |  |  |  |  |  |  |  |  |  |  |  |  |
| Pacific Islander/ Native Hawaiian |  |  |  |  |  |  |  |  |  |  |  |  |  |
| Missing/Unknown/Refused |  |  |  |  |  |  |  |  |  |  |  |  |  |
| **Ethnicity** |  |  |  |  |  |  |  |  |  |  |  |  |  |
| Non-Hispanic |  |  |  |  |  |  |  |  |  |  |  |  |  |
| Hispanic |  |  |  |  |  |  |  |  |  |  |  |  |  |
| Missing/Unknown/Refused |  |  |  |  |  |  |  |  |  |  |  |  |  |
| **US Federal Region†** |  |  |  |  |  |  |  |  |  |  |  |  |  |
| 1 |  |  |  |  |  |  |  |  |  |  |  |  |  |
| 2 |  |  |  |  |  |  |  |  |  |  |  |  |  |
| 3 |  |  |  |  |  |  |  |  |  |  |  |  |  |
| 4 |  |  |  |  |  |  |  |  |  |  |  |  |  |
| 5 |  |  |  |  |  |  |  |  |  |  |  |  |  |
| 6 |  |  |  |  |  |  |  |  |  |  |  |  |  |
| 7 |  |  |  |  |  |  |  |  |  |  |  |  |  |
| 8 |  |  |  |  |  |  |  |  |  |  |  |  |  |
| 9 |  |  |  |  |  |  |  |  |  |  |  |  |  |
| 10 |  |  |  |  |  |  |  |  |  |  |  |  |  |
| **Urban vs. Rural** |  |  |  |  |  |  |  |  |  |  |  |  |  |
| Rural/Highly rural |  |  |  |  |  |  |  |  |  |  |  |  |  |
| Urban |  |  |  |  |  |  |  |  |  |  |  |  |  |
| **BMI (kg/m^2^)** |  |  |  |  |  |  |  |  |  |  |  |  |  |
| <18.5 (Underweight) |  |  |  |  |  |  |  |  |  |  |  |  |  |
| 18.5 to <25 (Normal-weight) |  |  |  |  |  |  |  |  |  |  |  |  |  |
| 25 to <30 (Over-weight) |  |  |  |  |  |  |  |  |  |  |  |  |  |
| 30 to <35 (Obese I) |  |  |  |  |  |  |  |  |  |  |  |  |  |
| 35 to <40 (Obese II) |  |  |  |  |  |  |  |  |  |  |  |  |  |
| ≥40 (Obese III) |  |  |  |  |  |  |  |  |  |  |  |  |  |
| **Charlson Comorbidity Index (CCI)** |  |  |  |  |  |  |  |  |  |  |  |  |  |
| 0-1 |  |  |  |  |  |  |  |  |  |  |  |  |  |
| 2-3 |  |  |  |  |  |  |  |  |  |  |  |  |  |
| 4-5 |  |  |  |  |  |  |  |  |  |  |  |  |  |
| ≥6 |  |  |  |  |  |  |  |  |  |  |  |  |  |

* Adjusted for sex, age, race, ethnicity, geographical region, urban/rural location, BMI and CCI.

†Categorized according to the 10 Federal Regions drawn up by the Federal Emergency Management Agency: 1 (CT, MA, ME, NH, RI, VT), 2 (NJ, NY, PR), 3 (DC, DE, MD, PA, VA, WV), 4 (AL, FL, GA, KY, MS, NC, SC, TN), 5 (IL, IN, MI, MN, OH, WI), 6 (AR, LA, NM, OK, TX), 7 (IA, KS, MO, NE), 8 (CO, MT, ND, SD, UT, WY), 9 (AZ, CA, GU, HI, NV), 10 (AK, ID, OR, WA).

Table E. Trends over time in the associations (adjusted odds ratios*) of sociodemographic characteristics and comorbidity burden **with risk of SARS-CoV-2-related mortality** among all xxx million VA enrollees

|  | **Adjusted* Odd Ratio for SARS-CoV-2-related Mortality (95% CI)** | | | | | | |
| --- | --- | --- | --- | --- | --- | --- | --- |
|  | **Entire Period: February, 2020-March, 2021** | **February- April, 2020** | **May-June, 2020** | **July-August,**  **2020** | **September-October, 2020** | **November-December, 2020** | **January-February, 2021** |
| **Sex** |  |  |  |  |  |  |  |
| Female |  |  |  |  |  |  |  |
| Male |  |  |  |  |  |  |  |
| **Age  (years)** |  |  |  |  |  |  |  |
| 18-64 |  |  |  |  |  |  |  |
| 65-79 |  |  |  |  |  |  |  |
| ≥ 80 |  |  |  |  |  |  |  |
| **Race** |  |  |  |  |  |  |  |
| White |  |  |  |  |  |  |  |
| Black |  |  |  |  |  |  |  |
| Asian |  |  |  |  |  |  |  |
| American Indian/ Alaska Native |  |  |  |  |  |  |  |
| Pacific Islander/ Native Hawaiian |  |  |  |  |  |  |  |
| Missing/Unknown/Refused |  |  |  |  |  |  |  |
| **Ethnicity** |  |  |  |  |  |  |  |
| Non-Hispanic |  |  |  |  |  |  |  |
| Hispanic |  |  |  |  |  |  |  |
| Missing/Unknown/Refused |  |  |  |  |  |  |  |
| **US Federal Region†** |  |  |  |  |  |  |  |
| 1 |  |  |  |  |  |  |  |
| 2 |  |  |  |  |  |  |  |
| 3 |  |  |  |  |  |  |  |
| 4 |  |  |  |  |  |  |  |
| 5 |  |  |  |  |  |  |  |
| 6 |  |  |  |  |  |  |  |
| 7 |  |  |  |  |  |  |  |
| 8 |  |  |  |  |  |  |  |
| 9 |  |  |  |  |  |  |  |
| 10 |  |  |  |  |  |  |  |
| **Urban vs. Rural** |  |  |  |  |  |  |  |
| Rural/Highly rural |  |  |  |  |  |  |  |
| Urban |  |  |  |  |  |  |  |
| **BMI  (kg/m^2^)** |  |  |  |  |  |  |  |
| <18.5  (Underweight) |  |  |  |  |  |  |  |
| 18.5 to <25  (Normal-weight) |  |  |  |  |  |  |  |
| 25 to <30  (Over-weight) |  |  |  |  |  |  |  |
| 30 to <35  (Obese I) |  |  |  |  |  |  |  |
| 35 to <40  (Obese II) |  |  |  |  |  |  |  |
| ≥40  (Obese III) |  |  |  |  |  |  |  |
| **Charlson Comorbidity Index  (CCI)** |  |  |  |  |  |  |  |
| 0-1 |  |  |  |  |  |  |  |
| 2-3 |  |  |  |  |  |  |  |
| 4-5 |  |  |  |  |  |  |  |
| ≥6 |  |  |  |  |  |  |  |

* Adjusted for sex, age, race, ethnicity, geographical region, urban/rural location, BMI and CCI.

†Categorized according to the 10 Federal Regions drawn up by the Federal Emergency Management Agency: 1 (CT, MA, ME, NH, RI, VT), 2 (NJ, NY, PR), 3 (DC, DE, MD, PA, VA, WV), 4 (AL, FL, GA, KY, MS, NC, SC, TN), 5 (IL, IN, MI, MN, OH, WI), 6 (AR, LA, NM, OK, TX), 7 (IA, KS, MO, NE), 8 (CO, MT, ND, SD, UT, WY), 9 (AZ, CA, GU, HI, NV), 10 (AK, ID, OR, WA).
