# Supplementary material for: Changes in the associations of race and rurality with SARS-CoV-2 infection, mortality, and case fatality in the United States from February 2020 to March 2021: A population-based cohort study
Source: PLoS Med. 2021 Oct 21;18(10):e1003807. doi: 10.1371/journal.pmed.1003807 (PMC8530298; doi:10.1371/journal.pmed.1003807)
Supplement: Attachment — Submitted filename: REPLY TRENDS IN RISK FACTORS.docx [file pmed.1003807.s006.docx]

**Subject:** PMEDICINE-D-21-02587R1

**Dear Dr. Turner,**

**We would like to thank the Editors, the Statistical Reviewer and the external Reviewers for their extremely thoughtful comments that have allowed us to substantially improve the manuscript.**

**We also confirm that we have uploaded all Figures as individual TIF files with sufficient resolution.**

**Please see a point-by-point response below. Changes in the manuscript are noted in red font. In addition to the point-by-point response, please note the following major changes:**

a. **We added an analysis of trends in risk factors for case fatality as recommended by Reviewer #3, which resulted in two more tables (Tables 5 and 6)**
b. **We modeled age and BMI as restricted cubic splines with 5 knots as recommended by Reviewer #1, and extended the age distribution of the cohort from age≥40 years in the original paper to age≥18 in the current paper**

**Sincerely,**

**George N. Ioannou on behalf of all co-authors**
-----------------------------------------------------------

**A. Requests from the editors:**

**1. To your data statement, please add non-author contact details for those wishing to inquire about access to study data.**
Done

**2. Please add a study descriptor to the title following a colon, e.g., "...: a population-based cohort study".
Please quote the setting (e.g., "in the United States") in title and abstract.**
The title has been changed as follows, as suggested by the Editors:

“Changes in the associations of race and rurality with SARS-CoV-2 infection and mortality in the United States from February 2020 to March 2021: a population-based cohort study”

The abstract has been changed as follows:

“Background

We examined whether key sociodemographic and clinical risk factors for SARS-CoV-2 infection and mortality changed over time, in a population-based cohort study.

Methods and Findings

In a cohort of 7,627,983 persons enrolled in the United States Veterans Affairs healthcare system, we evaluated the independent associations of sociodemographic and clinical characteristics with SARS-CoV-2 infection (n=182,917) and mortality (n=8,886) at monthly intervals between February 1, 2020 and March 26, 2021”.

**3. Please remove the information on funding and competing interests from the title page. In the event of publication, this information will appear in the article metadata, via entries in the submission form.**
Done

**4. Please combine the "Methods" and "Findings" subsections of your abstract.**

Done

**5. To the new combined subsection, please add a new final sentence, which should begin "Study limitations include ..." or similar and should quote 2-3 of the study's main limitations.**
Done. We included the following sentences:

Limitations of the study include that changes over time in the associations of some risk factors may be affected by changes in the likelihood of testing for SARS-CoV-2 according to those risk factors; also study results apply directly to VA enrollees who are predominantly male and have comprehensive health care and need to be confirmed in other populations.

**6. Please quote aggregate demographic characteristics of study participants in the abstract.**
Done.

“VA enrollees had a mean age of 61 years (SD 17.7) and were predominantly male (90.9%) and White (64.5%), with 14.6% of Black race and 6.3% of Hispanic ethnicity”.
**7. In the abstract and throughout the paper, please quote p values alongside 95% CI, where available.**
Done

**8. We suggest beginning the "Conclusions" subsection of the abstract with "In this study, we found that ..." or similar.**
Done

**9. After the abstract, we will need to ask you to add a new and accessible "Author summary" in non-identical prose. You may find it helpful to consult one or two recent research papers in PLOS Medicine to get a sense of the preferred style.**
Done.

**10. In the Methods section, please state whether or not the study had a protocol or prespecified analysis plan, and if so attach the document(s) as a supplementary file, referred to in the text. Please highlight analyses that were not prespecified.**

The study did have a prespecified analysis plan. Our group usually prepares the analysis plan as a “draft manuscript” which includes a short Intro to frame the topic followed by the proposed Methods and analytic plan and ghost tables and figures for the results. This “draft manuscript” works well as an analysis plan especially for topics and datasets that we are very familiar with from prior research such as this one. We included this document as a Supplementary file.

The analyses that were not prespecified are mainly all the analyses recommended by the PLOS-Medicine reviewers and editors, including:

- The analysis of risk factors and trends in case fatality
- Modeling of BMI and age as cubic splines

We added the following statements in the Methods section:

Almost all analyses were proposed a priori as shown in the Supplemental Analytic Plan. Notable exceptions were the analyses of case fatality as an outcome and additionally modeling age and BMI as cubic splines, which were performed in response to reviewers’ comments.

**11. We suggest substituting "Black race", for example, with "Black ethnicity" throughout.**

We respectfully ask to retain the term “Black race” rather than “Black ethnicity”. This is because VA self-reported data specifically have options for “Black” under the race descriptions.

**12. Throughout the text, please format reference call-outs as follows; "... persons [8,9,14]." (noting the absence of spaces within the square brackets). In the reference list, please list no more than 6 author names, followed where appropriate by "et al.".**
Done

**13. Noting reference 5 and others, please ensure that references have full access details.**
Done (Ref #5 is still only Epub unfortunately)

**14. Please use the journal name abbreviation "PLoS ONE" in the reference list.**

Done

**15. Please include a completed checklist for the most appropriate reporting guideline, e.g., STROBE, as a supplementary file, labelled "S1_STROBE_Checklist" or similar and referred to as such in the Methods section.** **In the checklist, please refer to individual items by section (e.g., "Methods") and paragraph number, not by line or page numbers as these will generally change in the event of publication.**
Done.

Comments from the reviewers:

*** Reviewer #1:

**1. I confine my remarks to statistical aspects of this paper.  The general approach is fine, but I have one issue to resolve and some comments on the figures.** **The authors categorized BMI and age. Categorizing continuous variables is nearly always a bad idea. It increases type I and type II error and imposes arbitrary cutoffs. Here, I see why the authors did it - for ease of presentation of the tables and figures. But I'd like to see models with splines of those variables. You could then make graphs at several ages, to show how things change. So, e..g, the graph on the bottom of p. 18 could show age 18, 30, 40, 50 and 60 (or other ages - the authors could choose).**
Done.

In our multivariable models, we modeled age and BMI as restricted cubic splines with 5 knots corresponding to 5, 27.5, 50, 72.5 and 95 percentiles. We are presenting results at ages 25, 35, 45, 55, 65, 75 and 85 years and at BMIs of 20, 25, 30, 35 and 40 Kg/m^2^ (we could not produced results for BMI 15 or 45 due to too few events)

**For figure 1: First, this is a hidden dual axis graph and these are generally frowned upon. (It's dual axis because the scales are different, per the footnote).  What are the authors trying to show? If they want to show each series, they could use two line plots, stacked vertically. If they want to show the ratio of incidence and mortality, they could graph that. Yet another possibility is to have a scatter plot with mortality on one axis, incidence on the other, a dot for each time point, and a line  with arrows connecting the dots.**
We followed the Reviewer’s recommendations and plotted 3 plots stacked vertically as shown in Figures 1a-c.

We additionally now have results for case-fatality, as recommended by Reviewer #3

*** Reviewer #2:

**The analysis is presented in a manner that is clear, concise, and precise. The manuscript provides important findings about changing associations between race and odds of COVID-19 infection and mortality. These findings should be disseminated, after the authors address the following issues concerning their study. This should not require redoing the statistical analysis.

1.** **The Discussion section should provide greater depth and detail about how the VA population of minority groups are different from the general US population of these groups. Rates of chronic poverty (i.e., persisting over multiple years) and multigenerational poverty are elevated in the US African American population. Chronic, multigenerational poverty may result in health disparities for reasons outlined by Link & Phelan (1995) and their progeny. Is the VA population of African Americans as likely to have experienced this chronic disadvantage as African Americans in general? Are there other reasons that the VA population might consist of a relatively advantaged or privileged group of African Americans (versus the Black population in general), aside from the reason mentioned by the authors (i.e., greater access to healthcare)? The authors might look at whether Blacks in the VA population are more likely to have graduated high school, to currently own a home, or to currently not be in poverty than US Blacks in general.**
 **The authors should also address, relatedly, whether racial inequality within the VA population is analogous or parallel to racial inequality in the general population. Though this is not within my expertise, it is my understanding that there is much greater racial diversity among enlisted service members than among officers and especially upper-level officers. To what extent does this inequality in military rank parallel socioeconomic inequality between racial groups in the general population, whereby non-Hispanic Whites are more likely to have greater education and higher status/income jobs? How might differences in racial stratification within the military versus the general population affect differences in susceptibility to COVID-19 morbidity and mortality?**
We thank the Reviewer for these suggestions that have added nuance to our Discussion. We added the following paragraph in the Discussion to address and reflect the Reviewer’s comments:

Our comparisons of Black versus White VA enrollees need to be interpreted with caution when trying to extrapolate to differences by race in the US population as a whole. Black Veterans report higher median incomes ($44,000 vs. $26,000), lower unemployment (3% vs. 5%), and lower poverty levels (10% vs. 21%) than non-Veteran Black adults[27]. However, Black VA enrollees have lower socioeconomic status than White VA enrollees including much higher unemployment (22.5% vs. 6.8%)[28]. Levels of perceived discrimination in health care have been shown to be equally high among Black Veteran and non-Veteran groups in the US, despite access to comprehensive healthcare through the VA[29]. Furthermore, racial discrimination experienced during military service has been shown to be common among Black Veterans and associated with long-term impacts on self-reported physical health[30, 31]**.**

3.      **The authors do not control for educational attainment or other markers of socioeconomic status (SES) in their analysis. In the general US population, there are major differences between Blacks and Whites in educational attainment, poverty rates, and average income and wealth. In the analysis, race may thus be serving as a proxy for SES, and it may be the case that lower-SES individuals (regardless of race) were more likely to experience COVID morbidity and mortality. The authors could conduct a sensitivity analysis to determine whether results are substantially affected by controlling for an SES indicator, or their lack of controls for SES could be noted prominently as a limitation of the study requiring further exploration in later research.**
Unfortunately we do not have data on educational attainment or income. We acknowledge this as an important limitation now in the Discussion as follows:

We did not have data on educational attainment, poverty rates or income. It is possible that race or ethnicity may be serving as a proxy for low socioeconomic status; the extent to which the associations of race or ethnicity are confounded by socioeconomic status should be examined in future research.

4.      **Finally, I found the paper's presentation of its research methods to be somewhat confusing. If I am understanding it correctly, the paper provides results (in the tables) for separate regressions models run for monthly or bimonthly periods. The authors also state that they conducted a regression analysis with data combined from all periods, containing interactions between each predictor variable and a time-period ordinal variable. (This regression with the interaction terms is used—appropriately I believe—to test for change over time in the coefficients of the predictors.) The methods section is not explicit and clear enough in explaining that this single regression model with the interaction terms is distinct from the separate models for the different time periods, or that it is results from these separate regressions that are presented in the graph and tables. Likewise, I was looking for results in the form of a table for the combined regression containing the interaction terms, and I did not find them. This should be added to the paper, in the main text or the appendix.**
The Reviewer understands our Methods perfectly. We regret that our description was not clear.

We clarified in the “Statistical Analysis” section that indeed we used a separate multivariable logistic regression model for each monthly (or bimonthly) observation period to determine the associations between each risk factor and each of the three outcomes. This was clarified in the “Statistical Analysis” as follows:

“For each monthly observation period (for infections) or bimonthly observation period (for mortality and case fatality), we used a separate multivariable logistic regression model to simultaneously adjust for the 8 characteristics listed above to determine the associations between each risk factor and SARS-CoV-2 infection, mortality, or case fatality reported as an adjusted odds ratio.”

The Reviewer is also correct that we created a different model that combined all time periods and included an interaction term separately for each time period (risk factor*time period) to formally evaluate for statistically significant trends over time. The results of these interaction terms are shown in Supplemental Tables 1-2.

“We used a Wald test with cluster-robust standard errors to formally evaluate whether the associations between risk factors and each outcome (infection, mortality or case fatality) changed over time by creating another model that combined all time periods and included an interaction term separately for each risk factor (risk factor*time period) where time period was an ordinal variable (see **Supplemental Tables 1-2**)”.

***** Reviewer #3:**

This is a useful and important analysis drawing on a richly detailed data source. With that said, I think that the analysis 1. could take more advantage of the available data to give more insight into the drivers of disparity in infection and mortality and how these have changed over time, and 2. requires modification to the underlying statistical modeling to be suitable for publication. I have two major concerns related to these points:

**1. The authors' findings about the differential associations between sociodemographic variables and infections vs. mortality are difficult to interpret, as mortality from SARS-CoV-2 is necessarily contingent on infection. As a result, risk factors for mortality are necessarily correlated with those for infection. Since the authors are analyzing individual-level EHR data, rather than aggregated data, they are able to examine both risks of infection and case-fatality, i.e. the risk of death on infection.

Doing this is important as it would allow the specific contributions of factors such as the CCI to mortality to be understood in terms of their specific effects on infection and case-fatality. This would let us see, for example, whether the trend in mortality associated with increasing CCI reflects a stable case-fatality rate across CCI levels, versus some combination of changing infection risks (as evidenced in the relationship between CCI and infection), and case-fatality rates. Analyzing mortality and infection as separate outcomes acts as an unnecessary limitation on the analysis and is one the authors should rectify before the manuscript is suitable for publication.**

We have added completely separate analyses where the outcome is case fatality and are showing these in the new Tables 5 and 6. The study now has 3 outcomes, which collectively should present the complete picture:

a. SARS-CoV-2 infection
b. SARS-CoV-2-related mortality
c. Case Fatality
   Results of associations and trends in case fatality are described throughout the paper.

**2. The models included in the analysis employ a single parameter for each age group rather than allowing age-specific effects to vary as a function of race/ethnicity. A number of analyses have shown differential age-specific rates of SARS-CoV-2 infection by race/ethnicity, with older African-Americans experiencing much higher rates of infection than same-aged Whites in the early months of the pandemic. Consequently, adjusting for race using a single fixed effect, while not accounting for age x race/ethnicity interactions may result in biased estimates of both the group-specific effects and the associations between age, incidence, and mortality. The authors should provide updated results which include this interaction or show why it is not necessary (e.g. by using posterior predictions from the model which show good coverage of data as a function of age and race/ethnicity)**

Because our sample size is so large, almost all interaction terms are “statistically” significant. To evaluate whether the associations (and trends) between race and SARS-CoV-2 infection are different for older versus younger persons as the Reviewer suggests, we performed separate analyses stratified by age, i.e. in person aged <65 year or ≥65 years (see Supplemental Table 3 below). This analysis shows that the associations (and trends over time) between Black race and SARS-CoV-2 infection are actually remarkably similar in persons <65 year and those ≥65 years.

We added the following statement in the Results section and included the Supplemental Table 3 shown below in our Supplemental Tables:

“When we categorized our cohort by age (<65 and ≥65 years), the associations and their trends over time between Black (vs White) race and SARS-CoV-2 infection were very similar for persons aged <65 and ≥65 years (**Supplemental Table 3**)”.

**Supplemental Table 3. Trends over time in the associations (adjusted* odds ratios) of race with risk of SARS-CoV-2 infection presented separately for persons aged <65 versus ≥65 year among 9.1 million VA enrollees from February 2020 to March 2021.**

|  | **Adjusted* Odd Ratio for SARS-CoV-2 infection (95% CI)** | | | | | | | | | | | | | |
| --- | --- | --- | --- | --- | --- | --- | --- | --- | --- | --- | --- | --- | --- | --- |
|  | **Entire Period: February 2020-March 2021**  **N=**9,127,673 | **February- March 2020**  **N=**9,127,673 | **April**  **2020**  **N=**9,090,196 | **May**  **2020**  **N=**9,053,082 | **June**  **2020**  **N=**9,022,051 | **July**  **2020**  **N=**8,990,833 | **August**  **2020**  **N=**8,948,674 | **September 2020**  **N=**8,913,864 | **October**  **2020**  **N=**8,881,573 | **November 2020**  **N=**8,844,680 | **December**  **2020**  **N=**8,804,424 | **January**  **2021**  **N=**8,744,388 | **February**  **2021**  **N=**8,671,916 | **March**  **2021**  **N=**8,630,283 |
| **Age < 65 years** |  |  |  |  |  |  |  |  |  |  |  |  |  |  |
| **Race** |  |  |  |  |  |  |  |  |  |  |  |  |  |  |
| White | 1 | 1 | 1 | 1 | 1 | 1 | 1 | 1 | 1 | 1 | 1 | 1 | 1 | 1 |
| Black | 1.36 (1.34-1.38) | 4.05 (3.58-4.59) | 3.06 (2.83-3.31) | 2.77 (2.52-3.05) | 1.77 (1.65-1.90) | 1.86 (1.78-1.95) | 1.74 (1.63-1.86) | 1.36 (1.26-1.47) | 1.13 (1.06-1.20) | 1.02 (0.98-1.06) | 1.13 (1.09-1.16) | 1.25 (1.21-1.30) | 1.29 (1.23-1.36) | 1.14 (1.07-1.22) |
| Asian | 0.93 (0.89-0.98) | 1.75 (1.12-2.72) | 1.32 (0.97-1.79) | 1.15 (0.79-1.67) | 0.82 (0.64-1.06) | 0.94 (0.79-1.12) | 0.90 (0.69-1.17) | 1.02 (0.77-1.34) | 0.81 (0.65-1.02) | 0.83 (0.73-0.95) | 0.92 (0.84-1.02) | 0.98 (0.88-1.10) | 1.02 (0.86-1.20) | 0.81 (0.64-1.03) |
| AI/AN | 0.88 (0.83-0.94) | 0.59 (0.24-1.43) | 1.20 (0.82-1.75) | 1.74 (1.21-2.50) | 1.23 (0.96-1.58) | 0.92 (0.75-1.12) | 0.84 (0.62-1.12) | 0.99 (0.73-1.33) | 0.94 (0.76-1.17) | 0.88 (0.77-1.02) | 0.83 (0.73-0.94) | 0.83 (0.72-0.96) | 0.86 (0.69-1.06) | 0.68 (0.50-0.93) |
| PI/NH | 0.97 (0.92-1.03) | 1.15 (0.61-2.15) | 1.56 (1.12-2.17) | 1.40 (0.94-2.11) | 1.07 (0.83-1.39) | 0.96 (0.79-1.17) | 1.25 (0.97-1.61) | 0.84 (0.59-1.18) | 0.93 (0.73-1.19) | 0.86 (0.74-1.00) | 0.95 (0.85-1.07) | 0.96 (0.85-1.10) | 1.08 (0.89-1.31) | 0.76 (0.57-1.03) |
| Missing/  Unknown/  Refused | 0.87 (0.85-0.89) | 1.17 (0.91-1.50) | 1.13 (0.97-1.32) | 1.18 (0.99-1.42) | 0.88 (0.78-0.99) | 0.88 (0.81-0.96) | 0.93 (0.82-1.04) | 0.91 (0.80-1.05) | 0.84 (0.75-0.93) | 0.82 (0.77-0.87) | 0.86 (0.82-0.91) | 0.88 (0.83-0.93) | 0.86 (0.79-0.94) | 0.82 (0.73-0.93) |
| **Age ≥ 65 years** |  |  |  |  |  |  |  |  |  |  |  |  |  |  |
| **Race** |  |  |  |  |  |  |  |  |  |  |  |  |  |  |
| White | 1 | 1 | 1 | 1 | 1 | 1 | 1 | 1 | 1 | 1 | 1 | 1 | 1 | 1 |
| Black | 1.46 (1.43-1.49) | 6.57 (5.76-7.50) | 3.15 (2.93-3.39) | 2.34 (2.14-2.57) | 2.24 (2.06-2.45) | 1.98 (1.86-2.11) | 1.77 (1.64-1.92) | 1.29 (1.18-1.42) | 0.95 (0.88-1.04) | 1.06 (1.01-1.12) | 1.19 (1.14-1.24) | 1.27 (1.22-1.33) | 1.31 (1.23-1.39) | 1.25 (1.14-1.37) |
| Asian | 0.59 (0.54-0.65) | 1.59 (0.70-3.60) | 0.62 (0.34-1.12) | 0.62 (0.31-1.25) | 0.45 (0.26-0.78) | 0.75 (0.53-1.04) | 0.92 (0.60-1.41) | 0.94 (0.58-1.53) | 0.66 (0.41-1.05) | 0.47 (0.34-0.64) | 0.57 (0.47-0.69) | 0.52 (0.42-0.64) | 0.61 (0.45-0.83) | 0.51 (0.29-0.87) |
| AI/AN | 1.03 (0.96-1.10) | 2.10 (1.08-4.07) | 1.58 (1.08-2.32) | 1.28 (0.80-2.04) | 1.13 (0.77-1.66) | 1.00 (0.77-1.31) | 0.98 (0.70-1.38) | 1.02 (0.72-1.46) | 1.09 (0.85-1.40) | 1.03 (0.87-1.22) | 0.96 (0.83-1.12) | 0.98 (0.83-1.15) | 1.15 (0.91-1.44) | 0.61 (0.38-0.97) |
| PI/NH | 0.97 (0.90-1.04) | 0.61 (0.20-1.91) | 1.00 (0.65-1.54) | 1.26 (0.81-1.96) | 0.68 (0.43-1.08) | 0.92 (0.70-1.22) | 1.16 (0.84-1.60) | 1.52 (1.11-2.07) | 1.02 (0.77-1.36) | 1.14 (0.96-1.36) | 0.75 (0.63-0.88) | 0.99 (0.84-1.15) | 1.02 (0.80-1.29) | 0.82 (0.55-1.23) |
| Missing/  Unknown/  Refused | 0.73 (0.71-0.76) | 1.34 (0.98-1.82) | 0.93 (0.78-1.10) | 1.03 (0.85-1.25) | 0.72 (0.60-0.87) | 0.87 (0.77-0.97) | 0.73 (0.62-0.85) | 0.79 (0.67-0.93) | 0.73 (0.64-0.83) | 0.77 (0.71-0.83) | 0.71 (0.67-0.76) | 0.68 (0.63-0.74) | 0.67 (0.60-0.75) | 0.65 (0.55-0.77) |

* Adjusted for sex, age, race, ethnicity, geographical region, urban/rural location, BMI and CCI.

†Categorized according to the 10 Federal Regions drawn up by the Federal Emergency Management Agency: 1 (CT, MA, ME, NH, RI, VT), 2 (NJ, NY, PR), 3 (DC, DE, MD, PA, VA, WV), 4 (AL, FL, GA, KY, MS, NC, SC, TN), 5 (IL, IN, MI, MN, OH, WI), 6 (AR, LA, NM, OK, TX), 7 (IA, KS, MO, NE), 8 (CO, MT, ND, SD, UT, WY), 9 (AZ, CA, GU, HI, NV), 10 (AK, ID, OR, WA).
