# Supplementary material for: Changes in the associations of race and rurality with SARS-CoV-2 infection, mortality, and case fatality in the United States from February 2020 to March 2021: A population-based cohort study
Source: PLoS Med. 2021 Oct 21;18(10):e1003807. doi: 10.1371/journal.pmed.1003807 (PMC8530298; doi:10.1371/journal.pmed.1003807)
Supplement: Attachment — Submitted filename: FINAL REPLY TRENDS.docx [file pmed.1003807.s007.docx]

Dear Editors,

Thank you for the positive news! Very exciting!

Below, I address the remaining comments.

Regards,

George Ioannou

1. Noting "p<0.01" in your abstract, please quote exact p values or "p<0.001" throughout the paper.
Done

2. At the end of the abstract, please remove "even" (from "even reversed ...").
Done

3. The Author summary is quite "data heavy" whereas it is intended to provide an accessible summary of the paper's findings. Therefore, please remove the points quoting quantitative elements also present in the abstract, aiming for three subsections, each of about three points each. Please also avoid repetition in abstract and author summary.
Done

4. Please refer to the analysis plan and STROBE checklist by attachment name in your Methods section.
Done

5. Please use the journal name abbreviation "PLoS ONE" in the reference list.
Done

6. Please remove the competing interest information from reference 33, and any other relevant references.

Done
